# Drug-free in vitro activation and autologous transplantation in infertile women with diminished ovarian reserve: An experimental pilot study

**DOI:** 10.18502/ijrm.v22i6.16792

**Published:** 2024-08-05

**Authors:** Saghar Salehpour, Mahsa Kazemi, Sedigheh Hosseini, Nazanin Hajizadeh, Bahareh Karimi, Hajar Abbasi, Mitra Nemati, Samaneh Esmaeili, Hasti Ziaee

**Affiliations:** ^1^Department of Obstetrics and Gynecology, School of Medicine, Shahid Beheshti University of Medical Sciences, Tehran, Iran.; ^2^Department of Biology and Anatomical Sciences, School of Medicine, Shahid Beheshti University of Medical Sciences, Tehran, Iran.; ^3^Preventative Gynecology Research Center, Shahid Beheshti University of Medical Sciences, Tehran, Iran.; ^4^School of Medicine, University of Central Lancashire, Preston, United Kingdom.

**Keywords:** In vitro fertilization, Autologous transplantation, Infertility, Ovarian reserve.

## Abstract

**Background:**

Poor ovarian response and diminished ovarian reserves (DOR) significantly contribute to female infertility. Previous attempts have been made to enhance follicular growth and improve pregnancy outcomes in these participants.

**Objective:**

This study aimed to assess the efficacy of the in vitro drug-free activation technique of the ovarian reservation and in vitro fertilization stimulation cycle outcomes in DOR participants.

**Materials and Methods:**

This pilot phase study investigated the impact of in vitro activation (IVA) on ovarian reservation and in vitro fertilization outcome in 10 infertile women with DOR from May to December 2023 at Taleghani Infertility Center, Tehran, Iran. Participants underwent general surgery and laparoscopy, involving the removal of a portion of one ovary, immediate transfer to the laboratory, dissection into small cubes, and subsequent re-implantation into the cases's ovary. The primary outcomes, include the count of retrieved oocytes, the number of oocytes reaching metaphase, and the secondary outcomes were the quantity and the number of embryos transferred, implantation rate, and occurrence of clinical pregnancy.

**Results:**

The study revealed a significant increase in the antral follicle count before and after IVA (p = 0.033). Before IVA, the median estradiol level was 93.5 (57.0), which reduced to 79.0 (35.0) after IVA, indicating a statistically significant difference. On average, 2.3 (0.8) oocytes were retrieved, among which 1.5 (0.7) were metaphase II oocytes. The observed pregnancy rate among the 2 cases was 22.2%.

**Conclusion:**

The current study suggests that IVA may positively impact follicular growth and pregnancy outcomes among women with DOR.

## 1. Introduction 

In recent years, there has been an increase in the number of infertile women with diminished ovarian reserve (DOR). Various lifestyle risk factors, including smoking, alcohol consumption, poor diet, and specific medical treatments, significantly contribute to the observed upward trend of DOR (1). Responding poorly to standard ovulation stimulation treatments, these cases yield a limited number of low-quality antral follicles (2). In such cases, primary follicles exhibit difficulty in self-activation without external stimuli, hindering their growth to the primary stage and impeding the acquisition of mature eggs (3).

Numerous attempts have been undertaken to enhance follicular growth and improve pregnancy outcomes in these cases, but the outcomes have been disappointing. Traditionally, egg donation was the sole option, but emerging techniques aim to regenerate, rejuvenate, and activate ovarian germ cells. These methods encompass artificial gamete production from ovarian stem cells, intraovarian injection of activated platelet-rich plasma with calcium gluconate, autologous mitochondria transfer into oocytes, and androgen supplementation to heighten follicle sensitivity to external gonadotropin stimulation. However, routine implementation remains absent, and existing evidence is either weak or contentious (4). Primordial follicles in human ovaries face 3 fates: remaining dormant until needed, entering apoptosis directly from dormancy (resulting in the inability to produce normal mature eggs and early ovarian failure) or being selected for the growing follicle pool, thereby enhancing hormone secretion and ovulation. The balance between dormancy, death, and activation of these follicles is crucial for maintaining reproductive function (3).

Recent findings have indicated that drug-free ovarian cortex activation technology (drug-free in vitro activation [IVA]) can stimulate follicle growth and decrease ovarian reserve in cases with primary ovarian failure. This technique facilitates the activation of primary follicle pools in the resting state, potentially improving the likelihood of pregnancy (4).

The current study aimed to investigate the efficacy of the in vitro drug-free activation technique of the ovarian reservation and in vitro fertilization (IVF) stimulation cycle outcomes in DOR cases who referred to our infertility center from May-September 2023.

## 2. Materials and Methods 

### Study design and study participants

The current study was the pilot phase of an experiment investigating the effects of IVA on the ovarian reservation and outcome of the IVF cycle in 10 infertile women with DOR from May to December 2023, referred to Taleghani Infertility Center, Tehran, Iran. We followed the CONSORT 2010 guidelines for reporting (5).

The inclusion criteria encompassed individuals < 40 yr old, in POSEIDON group III or IV cases (5), with antral follicle count (AFC) < 5 and anti-Müllerian hormone (AMH) levels below 1.2 ng/dl, follicle-stimulating hormone (FSH) levels < 10 IU/L with a history of at least one failed stimulation cycle resulting in the inability to retrieve oocyte. Participants with male factor infertility and a positive history of hydrosalpinx or anatomical uterine disorders and endocrine disease were excluded from the study.

### Sample size

The sample size estimation was conducted using the formula below. Previous research indicated a drug-free IVA of follicles for infertility treatment in poor ovarian response participants with decreased ovarian reserve with a significance level ( α ) of 0.05, a power of 80%, and an equal proportion of sample size in both groups being compared, we determined a minimum sample size requirement of 10 participants in total (6, 7). 


$$N=2\times {\left(\frac{Z_{1-\alpha }+Z_{1-\beta }}{\delta_{0}}\right)}^{2}\times S^{2}$$

Baseline characteristics, including age, body mass index (BMI), cause of infertility, duration of infertility, AMH serum level, and hormonal profile like AMH, FSH, luteinizing hormone (LH), AFC, and estradiol (E2), were recorded before the surgery. Hormonal profile measurements were conducted on the second day of menses, while the count of pre-antral follicles was determined through sono-vaginal analysis following the IVA procedure. 3 months later, another series of hormonal level assessments with a blood test and AFCs on day 2 of the cycle by transvaginal ultrasound were performed. The associated data were recorded again during this phase.

### Intervention 

On the day of surgery, participants underwent general anesthesia. Through laparoscopy, a portion of ovarian tissue was extracted (each biopsy measuring approximately 5 x 5 x 3 mm). This tissue was promptly placed inside a sterile dish with media and transferred to the nearby laboratory. In the laboratory, within a sterile hood, the ovarian cortex was meticulously separated from the medulla under sterile conditions. A section of the cortex was forwarded to the pathology laboratory to ascertain the presence of any remaining follicles. Subsequently, the remaining cortex was promptly sliced into small cubes (measuring 1 x 1 x 1 cubic mm) using a scalpel blade. Approximately 15–20 of these pieces were carefully loaded into a transfer catheter containing minimal media and reintroduced into the ovarian incision (6). After 3 months, participants undergoing ovarian stimulation were administered the antagonist protocol using 150 units of FSH. The protocol for the study participants involved daily subcutaneous injections of recombinant FSH (150 IU/day), commencing on the second day of the menstrual cycle and continuing until the day of human chorionic gonadotropin (hCG) administration.

To prevent premature LH surges, they also received daily subcutaneous doses of 0.25 ganirelix, starting from day 6 of stimulation and continuing up to the day of hCG administration, daily subcutaneous dose of 300 IU of highly purified human menopausal gonadotrophin, starting from day 8 of stimulation and continuing until the day of hCG administration. The cycle was terminated, if no follicles of ≥ 11 mm were observed in ultrasound between 8 and 10 days of stimulation. Upon the ultrasound detection of at least 2 follicles reaching ≥ 18 mm, hCG administration at a dosage of 10,000 IU was initiated on the same day or the following day to prompt the final maturation of oocytes. Following 34–36 hr post-hCG administration, participants underwent oocyte retrieval, and intracytoplasmic sperm injection procedures were performed. Progesterone supplementation began on the day of oocyte retrieval.

ActogestⓇ 200 mg was administered daily intravaginally via suppository, while progesterone Amp, 50 mg/ml, was given intramuscularly on a daily basis. Embryo blast transfer took place on the 6 ${\;}^{\text{th}}$ day post oocyte retrieval, with a maximum of 2 oocytes transferred per participant. The quality of the transferred embryos was evaluated using the Istanbul consensus workshop criteria, previously detailed (8). Continuation of progesterone support persisted until the onset of menses or upon receiving a negative pregnancy test result. Participants underwent follow-up monitoring, during which hormone levels, follicular growth, and the overall ovarian response post-reimplantation were assessed. The associated data were recorded again during this phase. We also assessed follicular development and ovulation.

### Outcome ascertainment 

The primary outcomes included the count of retrieved oocytes and the number of oocytes reaching metaphase II, while the secondary outcomes were the quality and the number of embryos transferred, implantation rate (number of gestational sacs/n of embryo transferred), and clinical pregnancy rate with transvaginal sonography that was done by one expert infertility fellowship 5 wk after embryo transfer.

### Ethical considerations

The study protocol underwent review and approval by the Ethics Committee and Review Board of Shahid Beheshti University of Medical Sciences, Tehran, Iran (Code: IR.SBMU.RETECH.REC.1401.897) and is registered on the IRCT website on March 31, 2023, which has been last updated on May 26, 2024. Before the surgery, the study protocol was thoroughly explained to the participants, and each participant completed an informed consent form. Participants were also allowed to withdraw from the study at any phase.

### Statistical analysis 

We initially assessed the normality assumption using the Shapiro-Wilk test. Variables meeting the assumption were described using mean and standard deviation, while non-normally distributed variables were presented using median and interquartile range. Dichotomous variables were described using frequency numbers and proportions. Paired *t* tests or their non-parametric equivalent, the Wilcoxon test, were employed to assess pre- and post-IVA measurements. All statistical analyses were conducted using Stata software (Ver 17.0, College Station, Texas, USA). P-values < 0.05 were considered significant.

## 3. Results 

**Figure 1:**
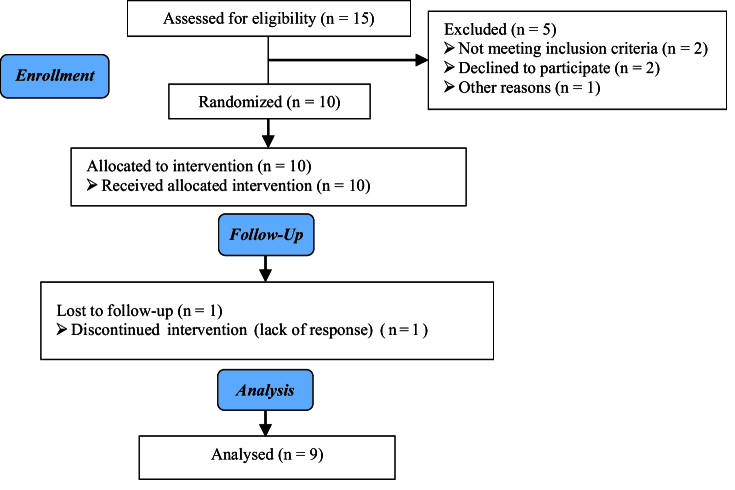
The study subjects' sampling flow chart.

A total of 15 participants were initially assigned to the study. However, 5 participants were subsequently excluded from the analysis: Not meeting inclusion criteria (n = 2), declining to participate (n = 2), and with other reasons (n = 1). 10 participants were allocated to intervention during follow-up, 1 cycle was canceled due to a lack of response, and finally, 9 cases were analyzed (Figure 1).

The study participants had a mean age of 36.8 yr with a standard deviation of 3.2 yr. The mean BMI was 25.1 ± 4.8.70% of participants presented with primary infertility, while 30% of cases exhibited secondary infertility after at least one spontaneous pregnancy. In all instances, the identified cause of infertility was attributed to female factors. The diagnosis of DOR was established according to the POSEIDON criteria groups 3 and 4. The mean duration of infertility was recorded at 2.2 ± 0.8 yr (Table I).

Table II provides a comparison of serum levels of menstrual hormones before and after IVA in the participants under investigation. The average serum level of AMH was 0.58 ± 0.28 ng/dl before IVA, decreasing slightly to 0.56 ± 0.30 ng/dl after IVA, with no statistically significant difference observed (p = 0.781). However, a significant increase in AFC was noted, with the average AFC rising from 1.75 ± 0.70 before IVA to 2.75 ± 0.70 after IVA (p = 0.033). Before IVA, the median E2 level was 93.5 ± 57.0 pmol/L, which reduced to 79.0 ± 35.0 pmol/L after IVA, and this observed difference was statistically significant (p = 0.002). Before IVA, the median LH and FSH levels were 13.41 ± 3.48 and 12.71 ± 3.60 pmol/L, which reduced to 12.07 ± 4.0 and 12.66 ± 3.91 pmol/L after IVA, and no statistically significant change was observed in the serum (p = 0.083 and 0.637) (Table II).

One cycle was canceled due to a lack of response and follicle growth on the 11 ${\;}^{\text{th}}$ day of the cycle. Among the other 9 participants, the average number of retrieved oocytes was 2.3 ± 0.8, with an average of 1.5 ± 0.7 being metaphase II oocytes. Embryo development was observed in 8 participants, while no embryos were formed for transfer in other cases despite obtaining 1 metaphase II oocyte. One cycle was canceled due to a lack of response and lack of follicle growth on the 11 ${\;}^{\text{th}}$ day of the cycle. Among the other 9 participants, the average number of retrieved oocytes was 2.3 ± 0.8, with an average of 1.5 ± 0.7 were metaphase II oocytes. Embryo development was observed in 8 participants, while no embryos were formed for transfer in another case despite obtaining 1 metaphase II oocyte (Table III).

The mean and standard deviation of the transferred embryos were 1.4 ± 0.8. In total, 13 embryos were transferred in 8 cases, in 3 of them a single embryo, and in the remaining 2 embryos were transferred, with BC quality showing the highest percentage among the transferred embryos. The proportion of embryos with B and C quality was 36.3% and 9.0%, respectively. The observed pregnancy rate across the 2 cases was 22.2 ± 2%. Implementation rate was also provided as the proportion of gestational sacs or fetal heartbeats observed during ultrasound (confirming a pregnancy) on the total number of embryos transferred, and it was 27.3 ± 3% (Table III).

The first pregnancy was observed in a 38-yr-old patient with a BMI of 32.4 kg/m². This patient experienced a 2-yr duration of infertility due to secondary causes. The baseline levels of AMH, FSH, E2, and LH were 1.0, 10.4, 54.6, and 19.0, respectively. Post-intervention, these levels reached 1.1, 10.0, 68.0, and 18.0, respectively. 2 oocytes were transferred, but only one proceeded to metaphasis, resulting in the development of a single embryo. The quality of the embryo was graded as BC. The second pregnancy occurred in a 36-yr-old woman with a slightly elevated BMI of 27.1 kg/m². The duration of infertility was 3.0 yr, attributed to a primary cause. At baseline, the levels of AMH, FSH, E2, and LH were 0.6, 12.1, 58.0, and 14.2, respectively. Following intervention, the hormone levels improved to 0.8 for AMH, 10.5 for FSH, 61.0 for E2, and 16.0 for LH. 2 oocytes reached metaphase, resulting in the development of 2 embryos. The quality of the transferred embryos was graded as B and BC. One pregnancy, which involved twins, progressed for 9 wk, but unfortunately, despite yolk sac and fetal pole formation, the heart did not form in the second case resulting in a 12-wk missed abortion.

**Table 1:** Study participant's baseline characteristics


**Characteristics**		**n = 9**
Age (Yr)*		36.8 ± 3.2
BMI (kg/m^2^)*		25.1 ± 4.8
Cause of infertility**		
	**Primary**	70 ± 0.7
	**Secondary**	30 ± 0.3
Duration of infertility (yr)*		2.2 ± 0.8
*Data presented as Mean ± SD. **Data presented as n (%). BMI: Body mass index		

**Table 2:** Follicular growth and serum hormone before and after drug-free IVA in participants with DOR and POI


**Hormone **	**Before IVA**	**After IVA**	**P-value**
AMH*	0.58 ± 0.28	0.56 ± 0.30	0.781
AFC*	1.75 ± 0.70	2.75 ± 0.70	0.033
E2**	93.5 ± 57.0	79.0 ± 35.0	0.002
LH*	13.41 ± 3.48	12.07 ± 4.0	0.083
FSH*	12.71 ± 3.60	12.66 ± 3.91	0.637
*Data presented as Mean ± SD, paired *t* test. **Data presented as Mean ± SD, Wilcoxon test. DOR: Diminished ovarian reserve, POI: Premature ovarian insufficiency, IVA: In vitro activation, AMH: Anti-Mullerian hormone, AFC: Antral follicle count, E2: Estradiol, LH: Luteinizing hormone, FSH: Follicle-stimulating hormone			

**Table 3:** IVF outcomes of the cases who underwent drug-free IVA


**Characteristics**		**n = 9**
N of oocytes*		2.3 ± 0.8
N of metaphase II oocytes*		1.5 ± 0.7
N of embryo transferred*		1.4 ± 0.8
Quality of transferred embryos**		
	**B**	5 ± 0.9
Quality of transferred embryos**		
	**BC**	7 ± 1.34
	**C**	1 ± 0.4
Number of gestational sacs/n of embryo transferred**		27.3 ± 3
Clinical pregnancy**		
	**Yes**	22.2 ± 2
	**No**	77.8 ± 7
*Data presented as Mean ± SD. **Data presented as n (%). IVF: In vitro fertilization, IVA: In vitro activation		

## 4. Discussion 

Our findings indicated an increased average number of AFC following IVA. Aging significantly diminishes the number of primordial follicles in the ovary, with a notable decline observed around the age of 40 (8). Although dormant follicles persist in participants with premature ovarian insufficiency and DOR, various attempts to regenerate, rejuvenate, or reactivate germ cells in the human ovary have not been successful (6).

Similar findings were reported demonstrating an increase in follicle growth among women diagnosed with DOR and poor ovarian response following drug-free IVA procedures. According to previous study, the observed growth in follicles and the increase in AFC were attributed to the presence of residual secondary follicles and their rapid growth post-IVA, as discussed. They refuted the role of AMH levels as a predictive factor in follicular growth post-IVA (9). Our findings were consistent with these prior results, as we observed no significant change in the levels of AMH before and after IVA. Meanwhile, one study argued that drug-free IVA only activates secondary follicles and does not affect primordial follicles. They explained this by suggesting that during drug-free IVA, the Hippo signaling pathway is disrupted, impacting the early stages of follicle development (10). Another study aimed to determine the clinical outcomes of utilizing the drug-free IVA technique for ovarian follicular activation. Their findings showed that this approach helped participants maintain their follicular waves for approximately 20 months post-surgery (2). Our study's pregnancy rate of 22.2% among participants with DOR who underwent the IVA procedure showcased a more favorable outcome compared to previous findings that reported a lower pregnancy rate of 15.3% in DOR participants who underwent traditional IVF (11). In study on women with DOR, the observed pregnancy rate was 31.0%, slightly higher than our current study. However, it is noteworthy that our study involved older women compared to study by Huang & colleagues study (12). Various studies have highlighted the significant influence of maternal age on IVF-related pregnancy outcomes, with younger women demonstrating a higher likelihood of achieving clinical pregnancy and live birth post-IVF (6, 12). While these findings lack confirmatory evidence due to the small sample size and absence of a comparison group, they serve as a potential stepping stone for further studies exploring the impact of drug-free IVA.

Our study marked one of the initial efforts to explore the clinical outcomes of drug-free IVA on follicular growth and pregnancy outcomes among women with DOR. Notably, our study was unique in its collection of data from 10 participants who underwent drug-free IVA, coupled with long-term participants follow-up to assess clinical outcomes, including chemical and clinical and ongoing pregnancies. However, it is essential to interpret our findings in light of certain limitations. The primary limitations of our study were the small sample size and the absence of a comparison group. Future clinical trials with randomization, larger sample sizes, and extended follow-up periods may offer deeper insights into both the benefits and potential adverse effects of drug-free IVA among women with DOR.

## 5. Conclusion 

In conclusion, the findings suggest that IVA may positively impact follicular growth and IVF outcomes among women with DOR, demonstrating a slight increase in AFC and pregnancy rates. Nonetheless, further trials involving larger sample sizes and comparison groups are essential to substantiate these findings and establish a clearer understanding, in this regard.

##  Data availability 

All supporting data are available through the corresponding author.

##  Author contributions

Saghar Salehpour and Nazanin Hajizadeh designed the study and conducted the research. Sedigheh Hosseini, Mahsa Kazemi, and Bahareh Karimi monitored, evaluated, and analyzed the results of the study. Further, Hajar Abbasi, Mithra Nemati, Samaneh Esmaeili, and Hasti Ziaee reviewed the article. All authors approved the final manuscript and take responsibility for the integrity of the data.

##  Conflict of Interest

The authors declare that there is no conflict of interest.
